# Is it necessary to alter anticoagulation therapy for tooth extraction in patients taking direct oral anticoagulants?

**DOI:** 10.4317/medoral.21942

**Published:** 2017-10-21

**Authors:** Mehmet Caliskan, Hüseyin-Can Tükel, Emre Benlidayi, Ali Deniz

**Affiliations:** 1 DDS, Oral and Maxillofacial Surgery, Faculty of Dentistry, Cukurova University; 2 DDS, PHD, Oral and Maxillofacial Surgery, Faculty of Dentistry, Cukurova University; 3 DDS, PhD, Department Head, Oral and Maxillofacial Surgery, Faculty of Dentistry, Cukurova University; 4 MD, Cardiology, Faculty of Medicine, Cukurova University

## Abstract

**Background:**

The number of patients using direct oral anticoagulants (DOACs) instead of vitamin K antagonists (VKA) is increasing and there is limited data on the safety of tooth extractions in patients taking DOACs. The aim of this study was to compare the amount of bleeding (AOB) and postoperative complications after tooth extractions between patients taking VKAs and patients taking DOACs without altering the anticoaguation therapy.

**Material and Methods:**

The study consisted of four groups: Direct thrombin inhibitor group, factor Xa inhibitor group, warfarin group and a control group. A single tooth was extracted in each patient and routine coagulation test values were recorded prior to extraction. AOB was measured for 20 minutes after tooth extraction. The patients were evaluated on 2nd and 7th days after extraction for bleeding. Status of bleeding was classified as no bleeding, mild bleeding controlled by gauze pads, moderate bleeding controlled by hemostatic agents and severe bleeding required hospitalization. Analysis of variance, chi square test and correlation analysis were used for statistical analysis of data.

**Results:**

A total of 84 patients (48 male, 36 female) were included in this study. The mean age of patients was 57 (38-87) years. Mean AOB was 1388.6±913.0, 1909.29±1063.1, 3673±1415.4, 1593.33±672.5 mg for direct thrombin inhibitor, factor Xa inhibitor, warfarin and control groups respectively. Mean AOB was significantly higher for warfarin group, compared to other groups (*p*<0.05). The number of patients showing mild and moderate bleeding was significantly higher in warfarin group compared to other groups on the 2nd postextraction day (*p*=0.001). No bleeding was occurred in control group on 2nd and 7th postextraction days and no bleeding was occurred in direct thrombin inhibitor group on 7th postextraction day. The number of bleeding events among groups was not statistically significant on 7th postextraction day (*p*=0.251).

**Conclusions:**

Patients taking warfarin had more bleeding compared to patients taking direct oral anticoagulants after tooth extractions. In patients taking direct oral anticoagulants simple tooth extractions can be safely carried out without altering the anticaogulant regimen with the use of local hemostatic agents.

** Key words:**Direct oral anticoagulants, dabigatran, rivaroxaban, apixaban, tooth extraction, oral surgery.

## Introduction

Treatment or prophylaxis of thromboembolism in several diseases and medical conditions including prosthetic cardiac valves, chronic atrial fibrillation, stroke, recurrent myocardial infarction, deep vein thrombosis and pulmonary embolism may require extended anticoagulation therapy. For more than 50 years VKAs have been the only oral anticoagulant drugs available (1). Warfarin is a coumarin derived drug that shows its anticoagulant effect by reduced synthesis of Vitamin K dependent factors II, VII, IX, X, proteins C and S. Warfarin is the most commonly prescribed VKA however it has many disadvantages like food and drug interactions, the need for individual dosing, regular monitoring and dose adjustment, slow onset and offset of action and a hypercoagulable state as a result of warfarin-mediated protein C and S deficiency that develops in some patients (2,3). To overcome these disadvantages and difficulties new oral anticoagulants or DOACs have been developed in the recent years (4). Unlike warfarin, DOACs act by inhibiting a specific targeted step in the coagulation cascade. The majority of DOACs including rivaroxaban(5) and apixaban (6) inhibit FXa. Dabigatran etexilate is the only available oral direct thrombin inhibitor for clinical use (7). The use of DOACs for thromboembolic disease treatment in place of VKAs has increased over the past years. The management of patients taking VKAs who require tooth extractions/oral surgery is well documented in the literature (8-14). However, there are only limited amount of clinical studies in patients taking DOACs who require dental extractions/oral surgery (15-18). The aim of this prospective, observational study was to compare the AOB and postoperative complications after tooth extractions in patients taking warfarin, direct thrombin inhibitors and factor Xa inhibitors.

## Material and Methods

Sixty-two patients taking oral anticoagulants and 24 healthy patients taking no anticoagulant were enrolled in this study. A medical history was taken, clinical and radiographic examinations were performed at first visit. Çukurova University Local Ethics Committee approved the study protocol and the guidelines established in the Declaration of Helsinki were followed during the study. The patients who participated in the study signed an informed consent.

The inclusion criterion was having a tooth indicated for extraction. Exclusion criteria were: local infection, inflammation or pathology associated with the tooth; conditions that may complicate the extraction including extensive caries, large restorations, roots that have severe dilaceration, ankylosis and hypercementosis; hematologic diseases; thrombocytopenia; hepatic/renal dysfunction; using an anti-platelet drug; INR>4 in patients taking warfarin.

The study was consisted of three anticoagulant groups and a control group; Patients taking direct thrombin inhibitor, patients taking factor Xa inhibitor, patients taking warfarin, patients taking no anticoagulants (control group). All patients in direct thrombin inhibitor group were taking dabigatran etexilate 150mg/12h (Pradaxa®, Boehringer Ingelheim Pharma GmbH & Co. KG). Three patients in FXa inhibitor group were taking apixaban 5mg/12h (Eliquis®, Bristol-Myers Squibb Co. & Pfizer) and 18 patients were taking rivaroxaban 20mg/24h (Xarelto®, Bayer Schering Pharma AG). The warfarin (Coumadin®, Zentiva, Istanbul) dosage was adjusted for a target INR value of 2.0 to 3.0 depending on the patient’s INR levels.

Blood samples were taken from all patients for BUN, creatinine, AST, ALT, prothrombin time (PT), INR and platelet count mea-surements on the day of extraction. Thrombin Time (TT) and anti-FXa measurements were performed for patients in direct thrombin inhibitor and factor Xa inhibitor groups respectively. When necessary, 2gr. amoxicilin were given to the patients for bacterial endocarditis prophylaxis 30-60 minutes before the extraction. A single tooth was extracted from each patient without stopping anticoagulants.

Extractions were performed under local anesthesia without epinefrine (3% Mepivacaine, Safecaine®, Vem Ilac, Istanbul, Turkey). Prior to extraction, the oral cavity was isolated from salivary secretions by specific saliva blocking barriers (Neodrys®, Henry Schein, Germany) placed on orifices of parotid glands, gauze pads placed on the submandibular gland ducts and by continuous suctioning with a surgical aspirator. All extractions were performed in an atraumatic fashion using elevators and forceps by the same surgeon who was blinded to the type of patient’s anticoagulant therapy. The method previously described by Karsli *et al.* was used to measure the AOB (10). At completion of the extraction, gauze pads were used to control bleeding from the extraction sockets, and these were subsequently changed for 20 minutes. Each gauze pad was placed over the extraction socket and changed with a new pad once it absorbed a sufficient amount of blood. The weights of gauze pads used before and after tamponade were measured using a fine electronic weight measurement device (Shimadzu, Kyoto, Japan). Weight differences before and after tamponades were interpreted as the AOB (in miligrams). After 20 minutes, each extraction socket was packed with oxidized cellulose dressing (Surgicel; Ethicon, Neuchatel, Switzerland) and sutured with 3.0 silk sutures (Dogsan, Istanbul, Turkey). Paracetamol 500 mg three times a day was prescribed for pain control. Patients were given additional gauze pads to be used if bleeding continued and instructed to call the surgeon or visit the oral and maxillofacial surgery department in case of severe bleeding. Patients were seen again on 2nd and 7th days after extraction for suture removal and evaluation of any postextraction bleeding. Status of postextraction bleeding was classified as no bleeding, mild bleeding controlled by gauze pads, moderate bleeding controlled by hemostatic agents and severe bleeding required hospitalization.

- Statistical analysis

All analyses were performed using SPSS 20.0 statistical software (SPSS, IBM, Chicago, IL). Categorical variables were expressed as numbers and percentages, whereas continuous variables were summarized as mean and standard deviation where appropriate. Chi-square test was used to compare categorical variables between the groups. For normal distributed data, One-Way ANOVA test was used to compare more than two groups. Bonferroni test was used for multiple comparisons of groups. Pearson’s correlation was used to assess the correlation between the AOB and TT in thrombin inhibitor group and between the AOB and Anti-FXa value in factor Xa inhibitor group. The statistical level of significance for all tests was considered to be 0.05.

## Results

**Figure 1:**
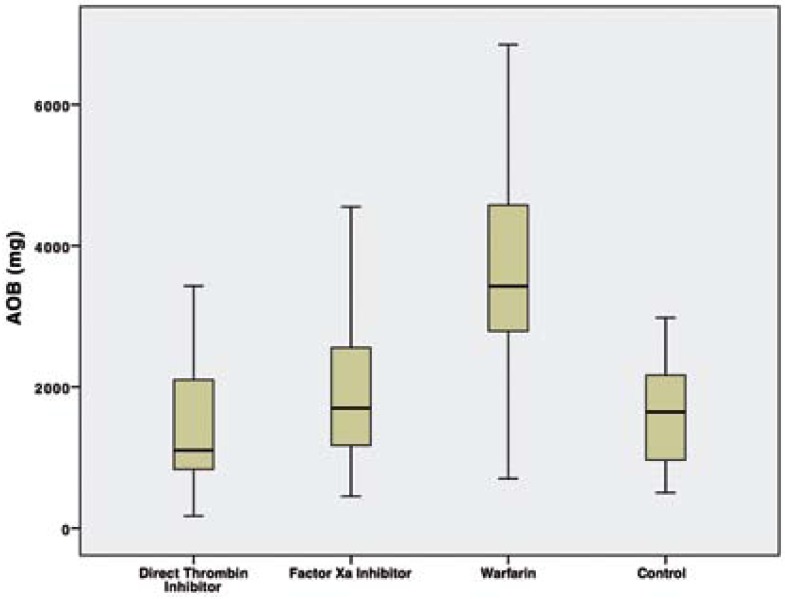
Graphic shows the AOB (mg) among groups.

Two patients taking direct thrombin inhibitor were excluded from the study because of elevated ALT-AST levels. A total of 84 patients (48 male, 36 female) were included in this study. The mean age of patients was 57 (38-87) years. Demographic data of the patients, type of extracted tooth and indication for anticoagulant therapy were summarized in  Table 1. There was no statistically significant difference among groups, in terms of age and type of extracted tooth (*p*>0.05). No patients required flap elevation, root separation or bone removal. No intraoperative complications or severe intraoperative bleeding occurred in any patient. The mean AOB after tooth extraction and bleeding status on 2nd and 7th postextraction days according to groups were summarized in  Table 2. The mean AOB in warfarin group was significantly higher than the other groups (*p*<0.05). The difference between thrombin inhibitor, factor Xa inhibitor and control groups was not statistically significant in terms of AOB (p1-2=0.798, p1-4=1.00, p2-4=1.00) (Fig. 1). No patient in control group had bleeding on 2nd and 7th postextraction days. No severe bleeding requiring hospitalization was encountered following the extractions. The number of patients showing mild and moderate bleeding was significantly higher in warfarin group compared to other groups on the 2nd postextraction day (*p*=0.001). No bleeding occurred on 7th postextraction day for thrombin inhibitor and control groups. One patient in factor Xa inhibitor group (4.8%) and 1 patient in warfarin group (4.5%) had mild bleeding, and 2 patients in warfarin group (9.1%) had moderate bleeding on 7th day. However these results were not statistically significant (*p*=0.251).  Table 3 shows the distribution of the laboratory results of the patients according to the groups. The mean INR and PT measurements were statistically significant between the groups (*p*<0.05). The direct thrombin inhibitor group and the control group were found to have similar INR and PT values. However, higher INR and PT values were measured in factor Xa and warfarin groups compared to direct thrombin inhibitor group and control group. On the other hand, no statistically significant difference was found between factor Xa and warfarin group (*p*=0.099). The mean platelet counts was not statistically significant among the groups (*p*=0.089). The mean TT in thrombin inhibitor group was 106.0±29.8 seconds, which was higher than normal range of TT (14-21 seconds). The mean anti-FXa was 2.98±9.2 UI/ml in factor Xa inhibitor group was also higher than the normal range (0-0.1 UI/ml). However no statistically significant correlation was found between the AOB and TT in thrombin inhibitor group (r=0.04; *p*=0.870), and the AOB and Anti-FXa value in factor Xa inhibitor group (r=-0.10; *p*=0.657).

**Table 1:**
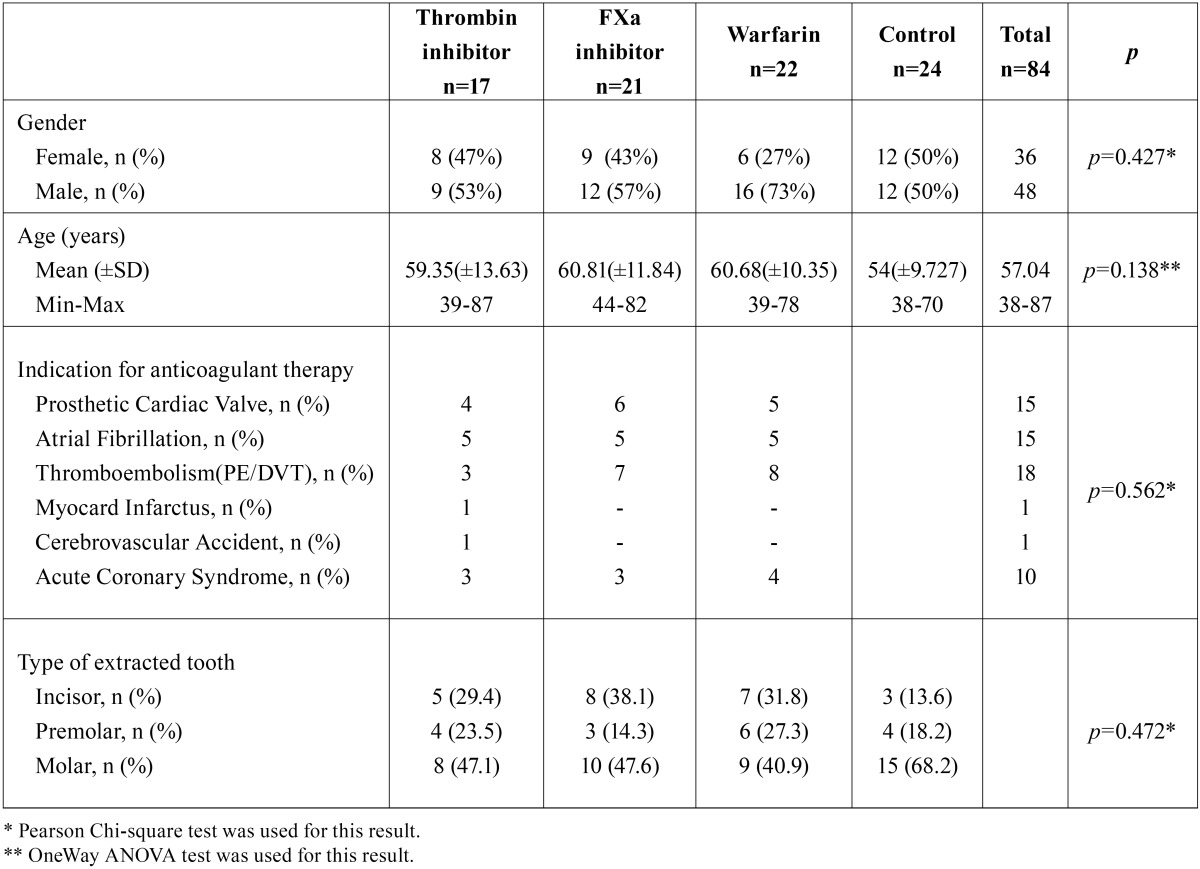
Patient characteristics, indications for anticoagulant therapy and type of extracted tooth.

**Table 2:**
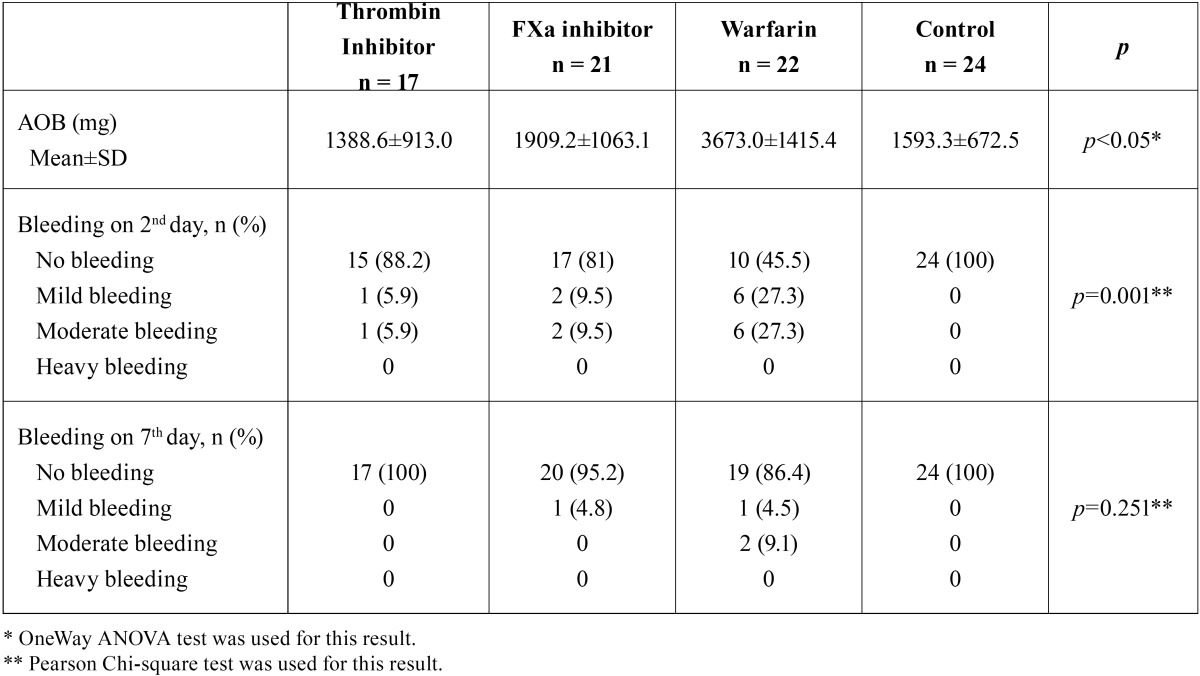
Amount of bleeding (AOB), bleeding on 2nd and 7th days after tooth extraction.

**Table 3:**
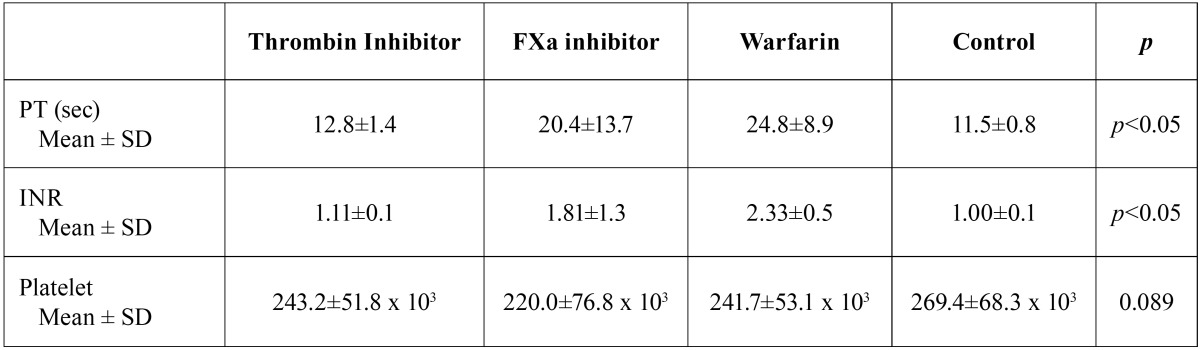
PT, INR and platelet count in groups.

## Discussion

Discontinuation of oral anticoagulants may lead to severe thromboembolic complications, and in case of minor surgery these medications should not be stopped if feasible. There is a considerable amount of literature that supports the continuation of VKAs in minor surgical procedures such as tooth extraction. Recent studies have shown that in patients taking warfarin, tooth extractions can be carried out safely with the aid of local hemostatic measures if the INR is less than 4 (11,14,19). Predictable pharmacokinetics, rapid onset of action, lower risk of food-drug interaction and a short half-life are advantages of DOACs compared to VKAs. Unlike VKAs, DOACs do not require regular monitoring or dose titration. Thus, management of patients taking these medications during tooth extraction may be safer and easier (3,20). However, a high level of scientific evidence for direct oral anticoagulant use during tooth extractions is limited. In a review by Muñoz-Corcuera *et al.* the authors suggest that each case should be treated individually in accordance to the risk of embolism, postoperative bleeding and renal function (21). Depending on the risk of bleeding Curto *et al.* classified dental treatments in two groups: procedures with a low risk of bleeding and procedures with medium to high risks of bleeding (22). Low-risk procedures include simple tooth extractions, oral surgery lasting less than 45 minutes and mucogingival surgical procedures. The extraction of more than three teeth at once and oral surgery lasting more than 45 minutes were considered medium-high risk procedures. In this group, the instructions to stop DOACs should be consulted with the specialist physician. The authors suggest that for low risk procedures discontinuation of dabigatran is not necessary and apixaban can be administered at a usual dose on the day, after the procedure (23). For medium and high risk procedures apixaban and dabigatran should be suspended for at least 24h and 48h respectively. Also the time of suspension should be extended for patients with altered renal function. If the drug suspension period is prolonged bridging therapy with low molecular weight heparin should be considered. Beyer-Westendorf *et al.* took data from a larger ongoing study of 2179 patients to evaluate the peri-procedural safety and management of DOACs(24). The majority of patients in the study were taking rivaroxaban (76%), followed by dabigatran (23.5%) and apixaban (0.5%). The authors defined three categories of procedures as minimal, minor and major according to the severity of tissue trauma and the risk of bleeding. In minor procedures category that included tooth extractions, the authors identified 3 (0.5%) major bleeding, 20 (3.1%) non-major clinically relevant bleeding and 6 (0.9%) minor bleeding. The prevalence of bleeding was found to be higher in the group of patients who had received heparin bridging, compared to patients continued DOACs or patients interrupted anticoagulants. In conclusion of the study heparin bridging was not recommended. In the present study, tooth extraction was performed without interruption of DOACs and no major bleeding was observed in any patient. Morimoto *et al.* extracted twenty-three teeth including two surgical extractions in 19 patients while continuing DOACs (17). The teeth were extracted in a minimally invasive fashion and the authors used local hemostatic measures after extraction. Three patients taking apixaban, and two patients taking rivaroxaban showed mild bleeding however one patient taking rivaroxaban who had a surgical extraction showed persistent post-operative bleeding on 2nd postextraction day. This patient had mild oozing from the surgical site even after local intervention using electrocautery and oxidized cellulose until the 7th day. No postoperative bleeding event was observed in other patients. Breik *et al.* reported a case series of 5 patients taking dabigatran who underwent tooth extraction (25). In one patient dabigatran was stopped 48 hours prior to extractions, in 3 patients a single tooth was extracted without drug interruption and there was no adverse bleeding in these 4 patients. However in one patient 18 teeth were extracted with drainage of a facial abscess under general anesthesia without drug interruption, which resulted in a significant bleeding. The patient needed to return to operating room and dabigatran was stopped to control the bleeding. Hanken et al. compared the postoperative bleeding events after 52 oral procedures including bone removal for tooth extraction and implant placement, performed while taking factor Xa inhibitor (rivaroxaban) with 285 oral procedures performed in patients taking no anticoagulant (18). The authors described postoperative bleeding event as a condition requiring additional treatment, which may correspond to moderate bleeding in our study. In this study 11.5% bleeding events incidence was reported for rivaroxaban, which was similar to our results for moderate bleeding (9.5%). The authors concluded that, although it is managable, continuing anticoagulation therapy with rivaroxaban significantly increases the risk of postoperative bleeding after oral surgery. Slightly increased incidence of bleeding events seen in this study may be explained by the bone removal for tooth extraction in the majority of the cases (97%). Mauprivez *et al.* compared the number of bleeding events after tooth extraction in 31 patients taking DOACs and 20 patients taking VKAs with an INR between 2.0 and 3.0 without interrupting their medications (15). The authors defined a bleeding event as persistent oozing or marked hemorrhage despite mechanical compression with gauze pads for 20 minutes. Five patients taking DOACs had seven bleeding events, and four patients taking VKAs had five bleeding events during the postoperative follow-up period. The difference between two groups was not statistically significant in terms of number of bleeding events. Eleven (91.67%) bleeding events were mild and controlled by gauze pads, and one (8.33%) was managed with a revision of the wound, application of fibrin glue, and resuturing. Similar to our study, the authors reported no bleeding that required hospitalization or blood transfusion. In contrast to our study no bleeding events were occurred after the first 3 postoperative days. Total bleeding events reported in this study (22.58%) was slightly higher than our results for thrombin inhibitor group and FXa inhibitor groups combined (18.42%). Two (9.52%) bleeding events were observed in 21 simple tooth extractions for patients taking dabigatran, which was similar to our study (11.8%). Eight (%29.63) bleeding events were observed in 27 simple extractions for patients taking Factor Xa inhibitor, which was slightly increased, compared to our results (23.8%).

In the present study the mean INR value was significantly higher in factor Xa inhibitor and warfarin groups compared to other groups. In patients taking warfarin, INR>2 is the therapeutic goal of anticoagulant therapy. Additionally, in patients taking factor Xa inhibitor an increase can be observed in INR. However, in patients using FXa inhibitor, there is no need for regular INR monitoring and a fixed dose is preferred. TT measured in the direct thrombin inhibitor group and anti-FXa measured in the FXa inhibitor group was higher than the normal ranges. However, no correlation could be found between these blood tests and AOB. Therefore, these tests are questionable for clinical practice.

The comparison of bleeding both quantitatively and in terms of postextraction bleeding events, the fixed dosage of DOACs and the prospective design were the main strengths of this study. It wasn’t possible to extract the same type of tooth from all patients and this could be considered as a weakness of this study. However the distrubution of types of tooth among the groups wasn’t different statistically. Perhaps a more important weakness of this study is that the time period between drug intake and tooth extraction was not recorded, which could be an important issue when considering the short half-life of these drugs. The most important limitation of this study was only a single uncomplicated tooth extraction was carried out for each patient. Multiple extractions or more invasive oral surgical procedures may show a different outcome and should be evaluated with further studies. Another limitation of this study was only 3 patients using apixaban was included in the study.

In conclusion, for simple tooth extractions DOACs are safe drugs in terms of bleeding and extractions can be carried out without interrupting or altering anticoagulant regimen with the aid of local hemostatic measures. Further studies are needed to evaluate the necessity of alteration in drug regimen for patients taking DOACs who underwent oral surgical procedures.
